# The complete mitochondrial genome of the freshwater crab *Potamiscus motuoensis* (Decapoda: Brachyura: Potamoidea)

**DOI:** 10.1080/23802359.2020.1720532

**Published:** 2020-02-03

**Authors:** Songbo Wang, Yifan Wang, Yangjin Zhuoma, Ling Tong, Zongheng Nie, Xinnan Jia, Chunchao Zhu, Xianmin Zhou, Jiexin Zou

**Affiliations:** a Research lab of Freshwater Crustacean Decapoda and Paragonimus, School of Basic Medical Sciences, Nanchang University, Nanchang City, Jiangxi Province, People’s Republic of China;; b Institute of Pathogen Biology, Jiangxi Academy of Medical Sciences, Nanchang City, Jiangxi Province, People’s Republic of China;; c Linzhi Municipal Center for Disease Control and Prevention, Linzhi City, Tibet Autonomous Region, People’s Republic of China;; d Medog County Center for Disease Control and Prevention, Linzhi City, Tibet Autonomous Region, People’s Republic of China;; e Key Laboratory of Poyang Lake Environment and Resource Utilization, Ministry of Education, Nanchang University, Nanchang City, Jiangxi Province, People’s Republic of China

**Keywords:** Brachyuran, complete mitochondrial genome, phylogenetic, *Potamiscus motuoensis*

## Abstract

*Potamiscus motuoensis* is the only one freshwater crab species distributed in Yarlung Zangbo River Grand Canyon and its complete mitochondrial genome was obtained for the first time. The complete mitochondrial genome of *P. motuoensis* is 17,971 bp in length, including 13 protein-coding genes, 22 tRNA genes, 2 rRNA genes, and 1 control region. In addition, the mitogenome has 19 noncoding regions ranging from 1 to 1396 bp in length. The report of the mitochondrial genome will enrich the species diversity of Yarlung Zangbo River Grand Canyon and provide data support for further research.

The species *Potamiscus motuoensis* (Crustacea: Malacostraca:Decapoda: Brachyura: Potamidae: *Potamiscus*) is the only one freshwater crab species distributed in Yarlung Zangbo River Grand Canyon.

An adult specimen of *P. motuoensis* was collected from Zhu Village, Damu Town Medog County, Linzhi City, Tibet Autonomous Region, China in 2014 (N29.523009° E95.432461°). The sample has been deposited in the Laboratory Specimen Library of Freshwater Crustacean Decapoda and Paragonimus, School of Basic Medical Sciences, Nanchang University, Nanchang, Jiangxi, China and National Parasite Germplasm Resources Specimen Library of China with a catalog number of NCUMCP4250. The sample was stored in 95% ethanol prior to extraction at room temperature before sequence analyses. Genomic DNA extraction, sequencing, gene annotation, and phylogenetic analyses were performed according to the method described by Plazzi et al. (Plazzi et al. 2013). The Bayesian Inference (BI) method was performed using MrBayes vers. 3.2 (Ronquist et al. 2012), with best model GTR + I+G selected by jModelTest vers.2.1.7. The maximum-likelihood (ML) method was performed using MEGA 6 (Tamura et al. 2013).

*Potamiscus motuoensis* (GenBank accession no. KY285013) has a full-length mitochondrial genome of 17,971 bp and shares the same 37 genes (13 protein-coding genes, 22 tRNA genes, and 2 rRNA genes) and 1 control region (CR) as the typical metazoan mitochondrial genome. Among these genes, 26 are located in the H-strand and 11 are located in the L-strand. The mitochondrial genome composition of *P. motuoensis* is A (35.5%), C (19.1%), G (9.2%), and T (36.2%), with a high A + T bias.

The mitochondrial genome of *P. motuoensis* contains 13 protein-coding genes. Similar to other Brachyura mitochondrial genomes, 9 protein-coding genes are located in the H chain (*COX1*, *COX2*, *COX3*, *ATP6*, *ATP8*, *ND2*, *ND3*, *ND6*, and *CYTB*), and the remaining four are located in the L chain (*ND1*, *ND4*, *ND4L,* and *ND5*). The initiator codon of most protein-coding genes is ATN (*ND4* is GTG), and the most frequent termination codon is TAA, containing incomplete termination codons TA and T and a rare termination codon TAG (*ATP8*). The A + T bias of the protein-coding gene is 69%. This bias is consistent with the genome.

The mitochondrial genome of *P. motuoensis* possesses 2 rRNA genes, *16S rRNA* and *12S rRNA*, which are located in the same L chain as other Brachyura mitochondrial genomes. The length of the *16S rRNA* and *12S rRNA* genes is 1294 bp and 859 bp, respectively; *16S rRNA* is between *tRNA^Leu(CUN)^* and *tRNA^Gln^*, while *12S rRNA* is between *tRNA^Gln^* and the CR. Similar to other Brachyura mitochondrial genomes, *P. motuoensis* has 22 tRNAs in common and most tRNAs have a typical clover structure, with the exception of *tRNA^Ser (AGN)^*, which lacks the dihydrouracil (DHU) arm (Ohtsuki et al. 2002).

The 22 tRNA genes are between 60 and 71 bp in length. Additionally, there are certain base mismatches, including 35G-T mismatches, 3A-C mismatches, 2A-G mismatches, 2T-C mismatches, 2A-A mismatches, and 2T-T mismatches. The mitochondrial genome of *P. motuoensis* has 19 noncoding regions and is between 1 and 1396 bp in length. The CR of *P. motuoensis* has the typical characteristic of crustaceans; it is located in a typical crustacean position (between *12S rRNA* and *tRNA^Val^*), is 859 bp in length and has a higher A + T bias than does the mitochondrial genome.

The phylogenetic position of *P. motuoensis* in mitogenome relative to other Brachyuran mitogenomes is determined by applying the BI and ML methods on 13 PCGs (Figure 1). The results were consistent with the current molecular classification and morphological classification (Segawa and Aotsuka 2005; Liang 2013; Ming et al. 2014; Tang et al. 2017), with other freshwater crab species of family Potamoidea forming a separate clade.

**Figure 1.**
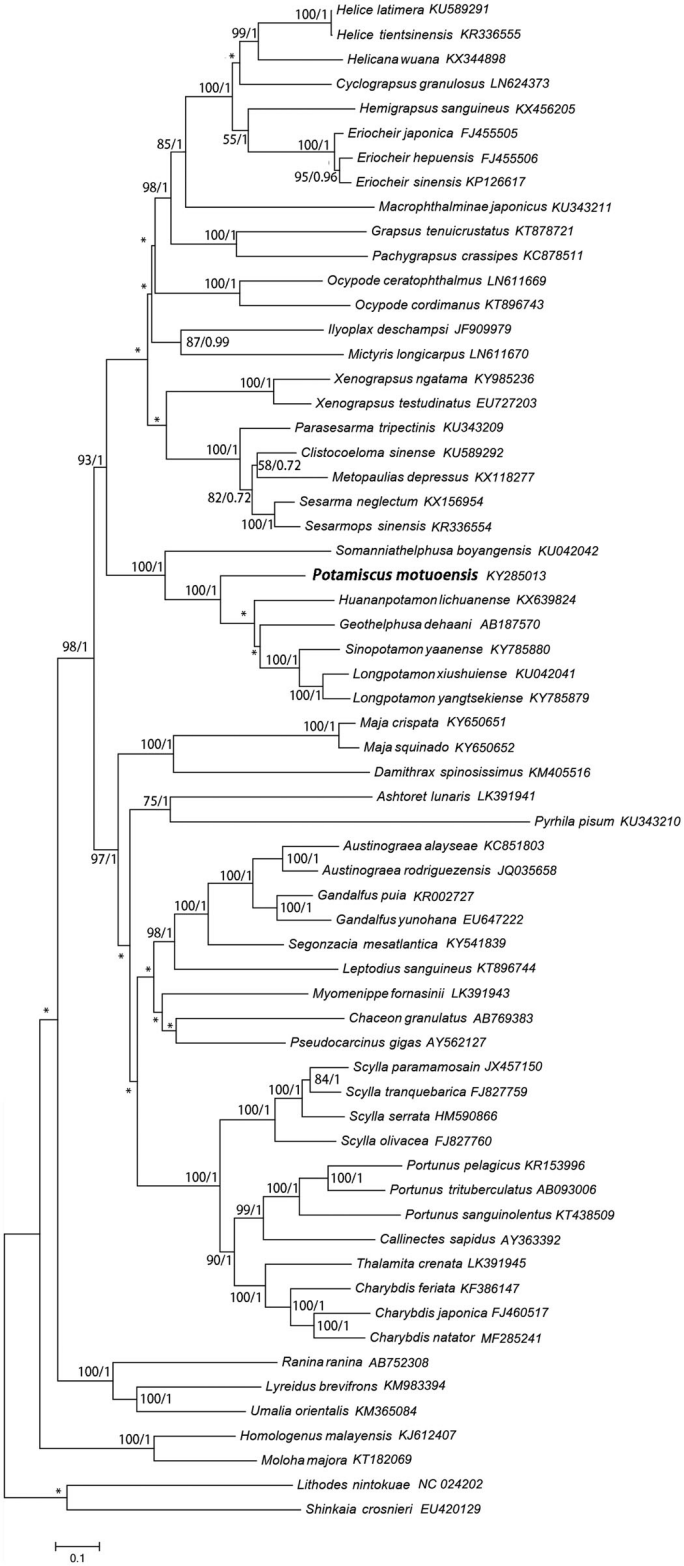
Phylogenetic maximum (ML) tree of *Potamiscus motuoensis* and related brachyurans based on 13 PCGs nucleotide sequences from the mitochondrial genome. *Lithodes nintokuae* and *Shinkaia crosnieri* serves as the outgroup. The numbers at the internodes are maximum likelihood (ML) bootstrap proportions and Bayesian inference (BI) posterior proportions. The differences between the ML and BI trees are indicated by ‘*’. The scale bars represent genetic distance.
